# The Transcriptome and Proteome of the Diatom *Thalassiosira pseudonana* Reveal a Diverse Phosphorus Stress Response

**DOI:** 10.1371/journal.pone.0033768

**Published:** 2012-03-29

**Authors:** Sonya T. Dyhrman, Bethany D. Jenkins, Tatiana A. Rynearson, Mak A. Saito, Melissa L. Mercier, Harriet Alexander, LeAnn P. Whitney, Andrea Drzewianowski, Vladimir V. Bulygin, Erin M. Bertrand, Zhijin Wu, Claudia Benitez-Nelson, Abigail Heithoff

**Affiliations:** 1 Biology Department, Woods Hole Oceanographic Institution, Woods Hole, Massachusetts, United States of America; 2 Department of Cell and Molecular Biology, University of Rhode Island, Kingston, Rhode Island, United States of America; 3 Graduate School of Oceanography, University of Rhode Island, Kingston, Rhode Island, United States of America; 4 Department of Marine Chemistry and Geochemistry, Woods Hole Oceanographic Institution, Woods Hole, Massachusetts, United States of America; 5 Department of Community Health, Center for Statistical Sciences, Brown University, Providence, Rhode Island, United States of America; 6 Marine Science Program, Department of Earth and Ocean Sciences, University of South Carolina, Columbia, South Carolina, United States of America; University of Minho, Portugal

## Abstract

Phosphorus (P) is a critical driver of phytoplankton growth and ecosystem function in the ocean. Diatoms are an abundant class of marine phytoplankton that are responsible for significant amounts of primary production. With the control they exert on the oceanic carbon cycle, there have been a number of studies focused on how diatoms respond to limiting macro and micronutrients such as iron and nitrogen. However, diatom physiological responses to P deficiency are poorly understood. Here, we couple deep sequencing of transcript tags and quantitative proteomics to analyze the diatom *Thalassiosira pseudonana* grown under P-replete and P-deficient conditions. A total of 318 transcripts were differentially regulated with a false discovery rate of <0.05, and a total of 136 proteins were differentially abundant (*p*<0.05). Significant changes in the abundance of transcripts and proteins were observed and coordinated for multiple biochemical pathways, including glycolysis and translation. Patterns in transcript and protein abundance were also linked to physiological changes in cellular P distributions, and enzyme activities. These data demonstrate that diatom P deficiency results in changes in cellular P allocation through polyphosphate production, increased P transport, a switch to utilization of dissolved organic P through increased production of metalloenzymes, and a remodeling of the cell surface through production of sulfolipids. Together, these findings reveal that *T. pseudonana* has evolved a sophisticated response to P deficiency involving multiple biochemical strategies that are likely critical to its ability to respond to variations in environmental P availability.

## Introduction

Phosphorus (P) supply is increasingly recognized as a major driver of marine ecosystems [1], influencing microbial genetic diversity [2] and global oceanic primary production [3]. Diatoms are an abundant and widespread class of phytoplankton, responsible for an estimated 40% of primary production in the ocean [4]. As such, they exert a profound influence on the global cycling of carbon. P concentrations are low in many marine systems, and there is growing evidence that P limits marine primary production in the subtropical North Atlantic [5], [6], and other major ocean systems [7], thus influencing the magnitude and efficiency of the carbon pump over modern and geological time-scales [3], [7]. In addition to their role in carbon export, diatoms are also important drivers of P export. A recent study by Diaz et al. (2008) suggests that polyphosphate stored in diatoms could also fall to the sea floor, exporting P and producing P-rich apatite-containing sediments [8].

P deficiency responses are well understood in model microbial eukaryotes like *Chlamydomonas reinhardtii* [9]. In non-model systems like marine phytoplankton, studies have identified changes in P uptake kinetics [10], [11], and the induction of alkaline phosphatase activity [10], [12], [13] as common strategies, to maximize phosphate uptake and hydrolyze phosphate from the typically larger pool of dissolved organic phosphorus (DOP) present in the upper water column of marine systems [14]. These strategies have only rarely been linked to a specific gene or protein [15]–[17]. There are an increasing number of studies that have used the two currently available marine diatom genomes (*Phaeodactylum tricornutum* and *Thalassiosira pseudonana*), to examine molecular level responses to macronutrient and iron deprivation [18]–[20] because of the importance of diatoms to the cycling of carbon in the ocean. Notably absent from these studies is a focus on P deprivation.

In this study, we conducted ultra-high throughput sequencing of transcript tags (tag-seq) [21], a type of digital gene expression (DGE), and quantitative shotgun liquid-chromatography mass spectrometry (LC-MS) of the proteome, to examine P deficiency responses in *T. pseudonana*. Transcriptome profiling with high throughput sequencing is increasingly the method of choice for studies in a wide range of organisms offering advantages over hybridization approaches [22]. Tag-seq is analogous to long-serial analysis of gene expression (Long-SAGE) [23] and generates 21-bp tags from the most 3′ NlaIII site of each transcript. With tag-seq, sequencing depth per transcript is high, because sequencing effort is focused at a primary location and is not distributed across each transcript as it is in other high throughput methods (e.g. RNA-seq [24]). Therefore, resolving power per sequencing effort on a given transcript is higher, yielding better statistical resolution of quantitative differences between samples [25]. LC-MS spectral counting was used to track changes in relative protein abundance between treatments, through counting and normalizing the number of spectra associated with each protein. This quantitative method is well-suited to examining changes in the proteome under differing environmental conditions, has not been previously applied to marine diatoms, and provides the basis for further quantitation of specific proteins on an absolute scale [26]. The joint application of global and quantitative transcriptome and proteome techniques to the study of nutrient deficiency in marine phytoplankton allows a comparison of the choreography between the transcriptome and proteome. Paired quantitative analyses of the transcriptome and proteome are still relatively novel in model organisms with well-characterized protein coding genes [27]–[29], and are not commonly applied to studies of environmentally relevant organisms, like marine phytoplankton, where the few available genome sequences lack widespread functional characterization. The data from the combined quantitative transcriptomic and proteomic profiling methods illuminate the global metabolic response of a diatom to P deficiency, with implications for diatom P physiology and the biogeochemical cycling of P in the ocean.

## Results and Discussion

### Quantitative transcript and protein profiling

P deficiency is a critical driver of phytoplankton dynamics in the ocean, but the molecular level drivers of cellular responses to changes in P supply are not well described in many marine phytoplankton. Herein, deep sequencing of transcript tags and quantitative proteomic analysis were performed on pooled biological replicates of P-replete and P-deficient cultures of *T. pseudonana* CCMP 1335 (Figure S1), and coupled with physiological measurements to elucidate biochemical responses to P deficiency.

To examine coverage of the 11,242 predicted protein coding genes [30], the unique tags were unambiguously mapped with 100% identity to the *T. pseudonana* CCMP 1335 genome, and those falling within a gene model were tabulated. Tag-seq, detects both sense and antisense transcripts. Here, tags frequently mapped to both the forward and reverse strands of a given NlaIII site (data not shown). It is unclear whether this is a function of true antisense transcription or a methodological issue [31], but the observation of antisense transcripts and their frequency was similar in a tag-seq study of *C. reinhardtii* [29]. For the purposes of the analysis presented herein, antisense tags were excluded. The data validate transcription of >85% (9,572) of the modeled gene set (Table S1; Table S2). Past transcriptional profiling in this diatom using a tiling array detected transcription of 4,653 (43%) of the predicted protein coding genes [19]. Tag-seq compares favorably with RNA-seq [29], and is of equivalent or better coverage than other tiling array, DGE, or SAGE studies [19], [32]–[34] with non-model organisms, like phytoplankton, where the transcriptome size is uncertain.

Differential expression patterns were determined on the sequence tags using Analysis of Sequence Counts (ASC) [25]. A total of 1382 differentially regulated sequence tags were detected in this dataset with an estimated false discovery rate <0.05 using ASC. Analysis of the 50 most significantly upregulated sequence tags in this set, identified 27 that could be assigned to annotated gene models (Table S3). Of the remaining tags, 15 mapped to the genome, and 8 could not be assigned to the genome (Table S3). Three of the 8 unassigned tags mapped to ESTs in the JGI genome portal and thus, may represent tags that map to unpredicted splice sites (Table S3). The remaining 5 tags map to neither the genome nor the ESTs, and likely represent tags that map to splice sites in genes with no EST support, or possibly tags that cover SNPs and do not map because of the requirement for a 100% identity match. Tags that did not align with gene models but aligned to the genome often matched intergenic spaces proximal to gene models associated with P metabolism, suggesting this is a function of tags mapping to the 3′ untranslated regions (UTRs). For example, the percentage of tags that map to gene models increases by roughly 24% when each gene model is extended 200 bp into the 3′ UTR. In several cases, tags align to the genome in a relatively isolated region of intergenic space (Table S3). These data suggest the presence of novel P-regulated genes that were missed by the *in silico* gene modeling and highlight the value of surveying tags that fall outside of the modeled gene set.

**Figure 1:**
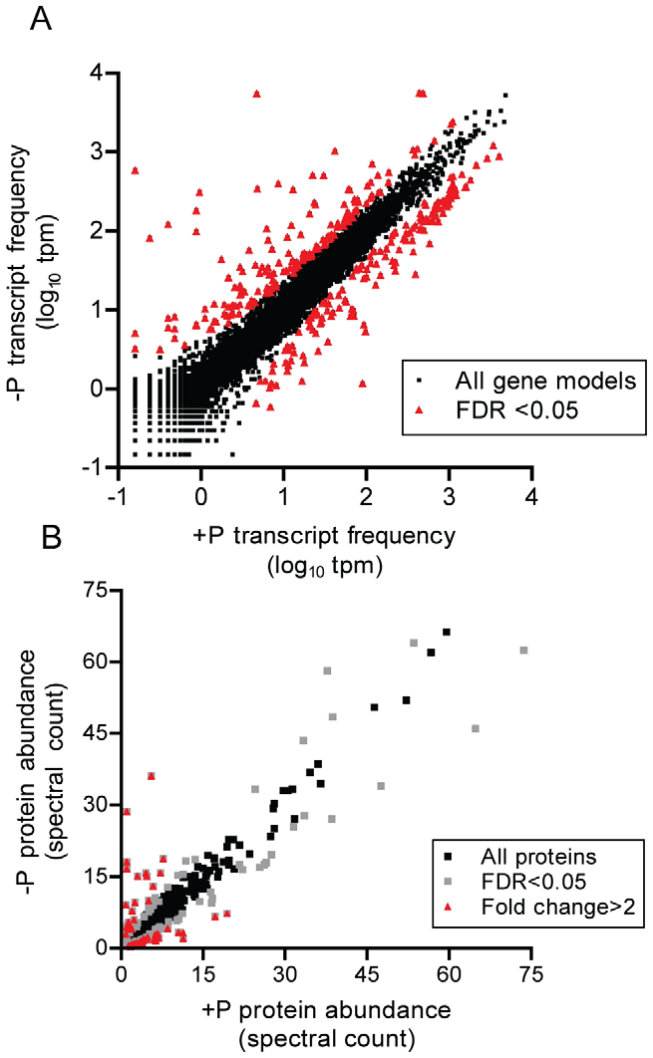
Phosphorus deficiency signals in the transcriptome and proteome. Global quantitative transcriptomic (A) and proteomic (B) analyses of *T. pseudonana* comparing P-replete (+P) and P-deficient (-P) treatments. Each data point represents a unique gene model (A) or protein (B), with those that are significantly differentially regulated noted in color. For the transcriptome data, tags mapped to a gene model were summed and then the total count was normalized to library size in tags per million (tpm). FDR is the false discovery rate p<0.05.

Differential expression patterns were also determined on transcripts with one or more sequence tags mapping to that gene model in the genome. Using ASC [25], a total of 318 differentially regulated transcripts were detected (Figure 1A; Table S1) with an estimated false discovery rate <0.05. The number is lower than for the raw sequence tags because there are multiple tags mapping to secondary NlaIII sites in the same gene model. Although the tag-seq method is designed to sample the most 3′ NlaIII site [22], mapping of tags to secondary sites within a gene model is common [29], [32], and likely related to incomplete digestion during library construction.

Quantitative global shotgun proteomic analyses were conducted in parallel to the transcriptome profiling, essentially providing independent replication of the regulation patterns observed in the transcriptome. Focusing on quantitative precision of the most abundant proteins, a total of 9512 unique peptides and 1264 unique proteins were detected with a false positive rate of 0.96% [35] (Table S4). Comparison of technical triplicate injections on the LC-MS demonstrated robust relative quantitation of the abundant proteins, with repeat injections showing similar spectral count values for each protein (Figure S2). The extent of protein coverage of gene products is similar to a study of the picoeukaryote *Ostreococcus tauri* [36] and a previous characterization of the *T. pseudonana* proteome [37], with the additional identification of certain P-responsive proteins (e.g. PID:23858) (Table S4). Changes in the relative abundance of proteins here were measured by spectral counting, where the number of spectra associated with a protein were counted and normalized. Using Fisher's Exact test (*p*<0.05), 136 differentially expressed proteins were identified (Figure 1B; Table S1). Of those, 79 were more abundant in the P-deficient treatment and 57 were more abundant in the P-replete treatment (Figure 1B; Table S1). On the order of 40 of the proteins were ≥2-fold more abundant in one of the conditions (Figure 1B). These observations show an increase in abundance of a number of transcripts and proteins that respond to an environmental stressor, and these targets may be useful for the development of biomarkers of diatom P deficiency in the oceans.

### Coordination in the transcriptome and proteome reveals diverse aspects of the P stress response

**Figure 2:**
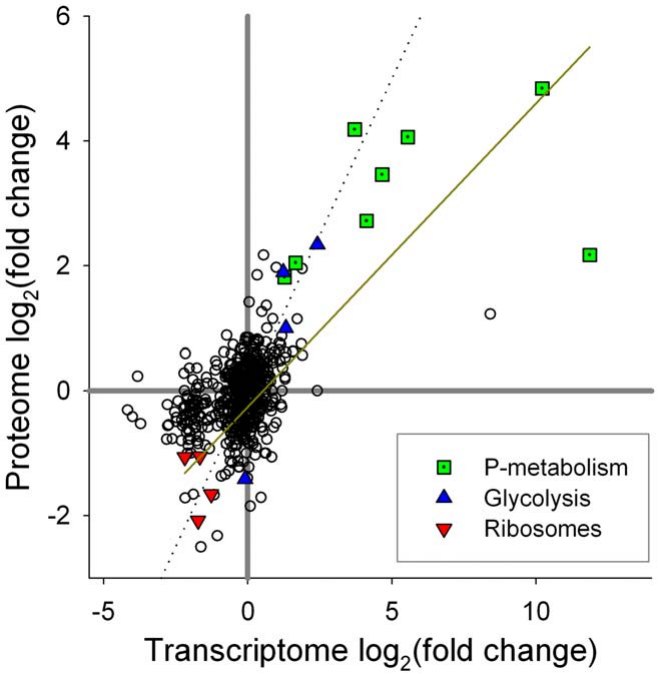
Comparison of transcript and protein signals. Comparison of proteome and transcriptome changes in response to P deficiency. Fold-change presented as the log_2_ of the ratio of deficient∶replete conditions. Unity lines are shown in grey solid (fold-change = 1), while a linear regression (log_2_[proteins] = 0.49*log_2_[transcripts]-0.25) of proteins that are >2-fold in abundance in either treatment against their corresponding transcripts is shown in yellow (r^2^ = 0.53). The dashed line is the 1∶1 line denoting equal fold change between the deficient and replete conditions for the transcriptome and the proteome. Proteins and transcripts of interest that correspond to P-metabolism, glycolysis and ribosomes/translation are highlighted.

A comparison of the transcriptome and proteome reveals a coordinated response for a number of targets, especially for those targets with a clear role in P metabolism (Figure 2; Table S5). In the combined dataset (where both the transcript and protein were identified for a given gene), 23 proteins were significantly more abundant in the P-deficient treatment, and 26 proteins were significantly less abundant in the P-deficient treatment (Table S5). Of the 23 proteins more abundant in the P-deficient treatment, no genes showed opposing patterns between the protein and the transcript. Coordination was also seen in the combined dataset for the 26 significantly downregulated proteins, with no transcripts showing opposing patterns (Table S5). Some transcripts were not significantly different between treatments, even though the corresponding protein was more or less abundant under P deficiency. This may be the result of post-transcriptional or post-translational regulation, or because they were expressed at low enough levels as to make it to difficult to resolve differences between samples. Of the few studies with model systems that have examined parallel responses of the proteome and the transcriptome in this way the extent of coordination between proteins and transcripts is similar. In yeast, 53% of proteins observed to be statistically more abundant had corresponding transcripts that were also statistically upregulated by rampamycin exposure [28]. In the green alga *C. reinhardtii*, 25% of transcripts responsive to copper exposure had a detectable protein with a coordinated pattern [29]. These studies have similar values to the diatom data examined here (60% for those that increased in abundance, 30% for those targets that were less abundant). With differences in the expression and turnover times for transcripts relative to proteins, and variability in post-transcriptional and post-translational regulation, there are many reasons why snapshot analyses of the transcriptome and proteome may not display similar patterns. Despite these possibilities, the patterns observed here for the diatom are tightly linked, particularly for the upregulated genes.

Analysis of the choreographed patterns, in combination with physiological data demonstrate that *T. pseudonana* has evolved a sophisticated, and multi-faceted, response to P deficiency involving five major strategies. These strategies include 1) change in cellular P allocation, 2) increased P transport, 3) a switch to utilization of DOP, 4) a remodeling of the cell surface, and 5) modulation of glycolysis and translation.

#### Changes in cellular phosphorus allocation

**Figure 3:**
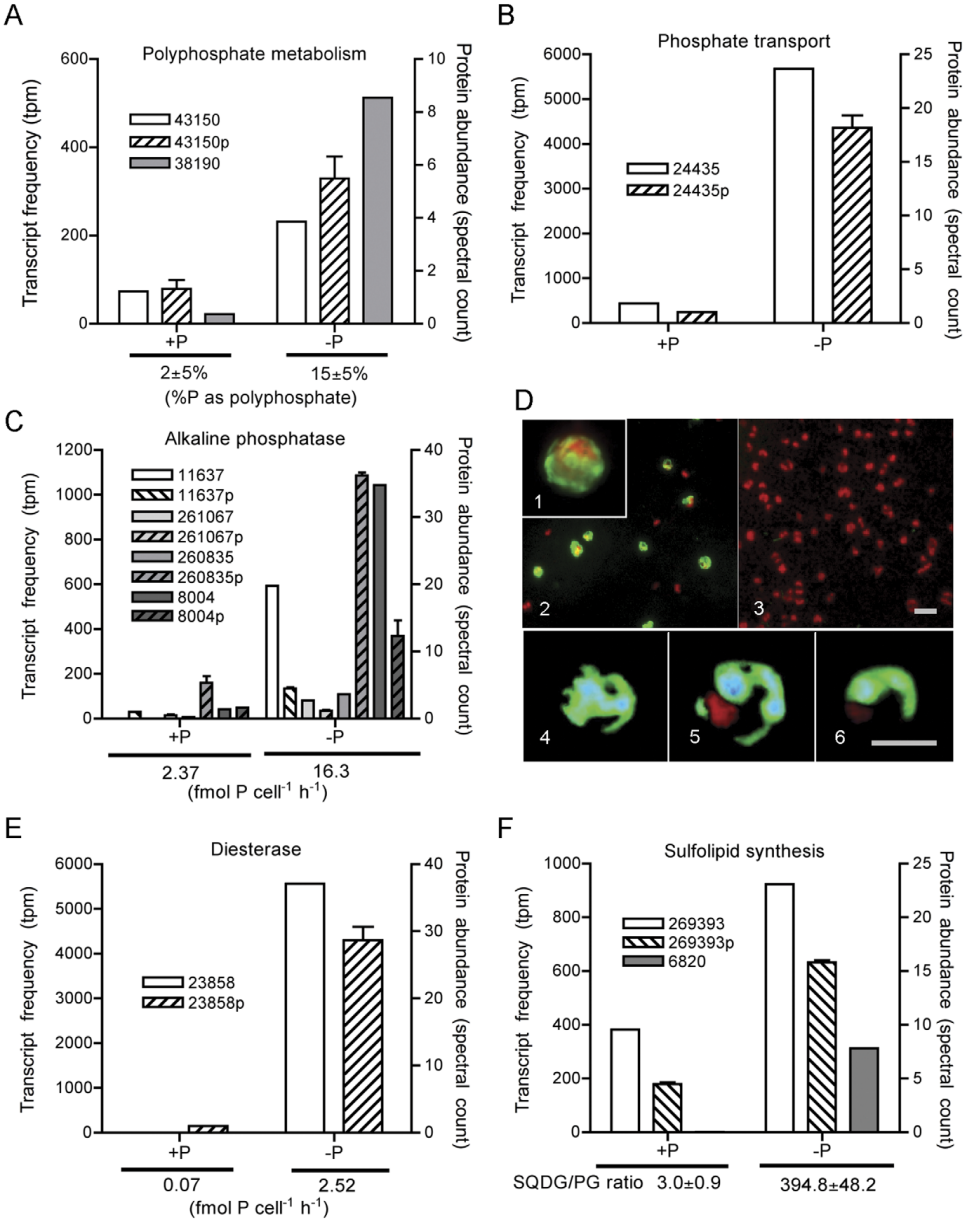
Transcript, protein, and physiological parameters linked to phosphorus deficiency. Normalized transcript and protein abundance for significantly differentially regulated signatures and their associated physiological patterns, for polyphosphate metabolism (A), phosphate transport (B), alkaline phosphatase (C, D), phosphodiesterase (E), and sulfolipid synthesis (F) across P-replete (+P) and P-deficient (-P) conditions. Protein data are distinguished with a “p” next to the PID and by the hatched pattern. Polyphosphate abundances as measured by solid state ^31^P NMR (A) and enzyme activities (C, E), were assayed and are reported below each graph. Cell-associated alkaline phosphatase activity (green color) was detected using an enzyme labeled fluorescence substrate. The green fluorescence indicating enzyme activity is present in -P cells (panels 1, 2, 4, 5, and 6) and not present in +P cells (panel 3) (D). Chlorophyll autofluorescence (red) in also visible. Panels 4, 5, and 6 are a Z series through a labeled –P cell. The SQDG:PC ratio is reported from Van Mooy et al. (2009) from replete and P-deficient *T. pseudonana* cultures [51], which were grown similarly to those in this study. SQDG: sulphoquinovosyldiacylglyerol; PG: phosphatidylglycerol.

Polyphosphate is comprised of variable length chains of phosphate, and it can serve as a storage compound that accumulates during luxury uptake in P-replete environments. Polyphosphate metabolism in yeast and other eukaryotes involves the vacuolar transporter chaperone (Vtc) 1–4 family [38]. In yeast, the Vtc4 protein interacts with the vacuole membrane and generates polyphosphate from ATP in a phosphotransfer reaction to form polyphosphate chains [39]. Two putative *T. pseudonana* Vtc genes were differentially regulated in this study. A Vtc4 polyphosphate polymerase homolog (PID: 43150) and an additional Vtc gene (PID: 38190) were significantly upregulated in the P-deficient transcriptome (Figure 3A). The VTC4 protein was also more abundant in the P-deficient proteome (Figure 3A). The other Vtc gene (PID: 38190) was not detected in the proteome, either because it is in low abundance, or because Vtc proteins are membrane associated and difficult to extract. Modulation of polyphosphate stores can occur in both prokaryotic and eukaryotic microbes experiencing general stress among other factors [40]. Although *T. pseudonana* is known to increase P allocation to polyphosphate in response to nitrogen limitation in chemostat cultures [10], polyphosphate in marine systems is typically thought to be the product of luxury uptake and storage of P in phytoplankton [8], not a stress response. Herein, the gene expression and protein abundance patterns are consistent with an increased allocation of P to polyphosphate detected in P-deficient *T. pseudonana* with solid state ^31^P NMR (Figure 3A). These data emphasize that not all diatom polyphosphate allocation is driven by luxury uptake in high P coastal systems, and thus supports the hypothesis that polyphosphate is present and its cycling could be important in low P systems [41], and in scenarios where diatoms are subject to environmental stressors. With these novel regulation data for a diatom polyphosphate polymerase, and their link to cellular polyphosphate changes, the transcript or protein may be used to track polyphosphate dynamics in field populations and could serve as a tool for better constraining patterns in P allocation, polyphosphate export, and concomitant apatite formation in marine sediments.

#### Increased phosphate transport

A common response to P deprivation is to increase phosphate transport kinetics, either by inducing transporters with higher affinity (change in K_m_), or making more transporters, which can result in an increase in maximal uptake (V_max_) with increasing P-limitation of growth rate [11]. In a study by Perry (1976), *T. pseudonana* P deficiency induced a dramatic increase in V_max_, but no change in K_m_ [10], suggesting the induction of a P transporter, but not a high affinity one. Consistent with this observation, a phosphate transporter (PID:24435) is upregulated in both the transcript data and protein data under P deficiency (Figure 3B). In fact, the transcript had the highest absolute tag counts in the P-deficient dataset (Table S2). This is consistent with changes in P uptake capacity observed in *T. pseudonana*, and now links rate changes in P uptake to this gene. Shifts in uptake kinetics are rarely incorporated into marine biogeochemical models [42], but are likely an important area where transcriptomics and proteomics can influence marine biogeochemical modeling. Future work that confirms similar responses in other phytoplankton species and that titers transporter number to uptake rates over a range of substrate availability would offer a mechanism to address this gap between physiology and predictive power.

#### Utilization of organic phosphorus sources

Genes for several putative alkaline phosphatases (PID: 11637, 261067, 260835, and 8004) are upregulated under P deficiency in both the transcriptome and the proteome (Figure 3C). The induction of these alkaline phosphatases is associated with increased alkaline phosphatase activity (Figure 3C). This enzyme has a broad substrate specificity for phosphomonoesters, and *T. pseudonana* is able to grow on exogenous phosphomonoester as a sole P source in axenic cultures (Figure S1). Alkaline phosphatase activity is critically important in the processing of marine DOP, which can support primary production [5]. The presence of multiple alkaline phosphatases has been predicted in other marine eukaryotic phytoplankton, based on kinetic patterns and substrate specificity [11], [43], but this has not routinely been linked to the expression of multiple alkaline phosphatases, only single candidate genes [15]. The redundancy observed here for a diatom may cover different substrate specificities, cellular localizations, or the metal requirements of the enzyme.

The majority of alkaline phosphatases detected in the P-deficient condition, have no clear signal peptide, however PID 11637 and 8004 appear to be secreted, and we hypothesize they are localized to the cell-surface. Although a recent study on marine bacteria suggests the presence and even dominance of extracellular alkaline phosphatases that are likely released to seawater [44], the alkaline phosphatase activity herein was detected on whole *T. pseudonana* cells (Figure 3D) with enzyme labeled fluorescence [45]. This activity appears to be surface-associated, with some localized intracellular labeling (Figure 3D). The fact that *T. pseudonana* can grow on phosphomonoester as a sole P source (Figure S1) is also consistent with at least one or more of the alkaline phosphatases being surface-associated. The simultaneous upregulation of both a putative phosphate transporter (Figure 3A) and a surface-associated alkaline phosphatase (Figure 3C, D) suggests that hydrolysis and uptake may be tightly coupled in this diatom and perhaps other phytoplankton.

In addition to the alkaline phosphatases, there is a 5′ nucleotidase (PID: 38194) upregulated in both the transcript and protein datasets (Table S5). This would allow cells to hydrolyze phosphate from nucleotides. There is no clear localization signal for this transcript, and the extent to which this gene product is involved in the processing of intracellular or exogenous nucleotides cannot be resolved. 5′ nucleotidase activity is not typically regulated by P in marine prokaryotes [46], but the expression data herein is consistent with the P regulation of 5′ nucleotidase activity that has been observed in other eukaryotic phytoplankton [43].

The majority of studies on DOP utilization have focused on phytoplankton use of phosphomonoester substrates. Although phosphoester dominates marine DOP [47], diester is rarely resolved from monoester in many of these characterization studies, and may be a larger portion of the DOP ester pool than is typically appreciated. In this study, a putative glycerophosphoryl diester phosphodiesterase (PID: 23858) was present at 5560 tpm in the P-deficient transcript dataset, and only 5 tpm in the P-replete dataset (Figure 3E). This massive upregulation and concurrent high relative protein abundance (roughly 20 fold more abundant in the P-deficient treatment) suggests the importance of this enzyme to *T. pseudonana* cells experiencing P deficiency, and provides new insight into the nature of P stress responses in phytoplankton. The enzyme typically displays a broad specificity for glycerophosphodiesters, and in bacteria they can be P-regulated, where they are involved in the processing of P from exogenous phosphodiester, or deacylated phospholipids [48]. Studies of diesterase activity in marine phytoplankton are rare, but the diatom *Chaetoceros ceratosporus* induced diesterase activity with P deficiency, whereas the diatom *Skeletonema costatum* did not [49]. The diesterase activity detected in *T. pseudonana*, is clearly also P-regulated in conjunction with the transcript and protein (Figure 3E). The signal peptide indicates the protein may be secreted, it and could be localized to the outer membrane to function in the hydrolysis of exogenous phosphodiester or in the recycling of P from lipid phosphodiester such as, glycerophosphocholine, and glycerophosphoglycerol, which are known to rapidly decline under P stress in diatoms [50].

#### Remodeling of the cell surface

Recent work has highlighted that *T. pseudonana*, and other phytoplankton, will conserve P in low P medium by replacing P containing lipids with non P containing sulfolipids (sulfur containing) and betaine lipids (nitrogen containing) [51]. This ability to adjust cellular P quota is an important adaptation to oligotrophy, allowing cells to conserve P by both adjusting the P quota, and to recycle lipid P [50]. The gene pathways that drive this switch have not been characterized in any marine eukaryotic phytoplankton, but sulfolipid sulfoquinovosyldiacylglycerol (SQDG) biosynthesis protein (*sqdX*) [52] and a UDP-sulfoquinovose synthesis protein (*sqdB*) [53] are known to be involved in sulfolipid biosynthesis, and *btaA* and *btaB* are known to be required for synthesis of betaine lipids in bacteria [54]. This process appears similar in the green algal lineage, although there is a fusion of *BtaA* and *BtaB* found in *R. sphaeroides* to form BTA1, a betaine lipid synthase, which controls betaine lipid biosynthesis in *Chlamydomonas* [55]. *T. pseudonana* has a clear homolog for *sqdB* (PID: 269393) and a related sulfolipid SQDG biosynthesis protein (PID: 6820). Both are upregulated in the P-deficient transcriptome (Figure 3F). The more highly expressed of these two genes (PID: 269393) is also more abundant in the P-deficient proteome (Figure 3F), and occurs in conjunction with a change in the ratio of SQDG to the phospholipid phosphatidylglecerol observed previously in P-deficient *T. pseudonana* [51]. There is not a clear homolog of either BTA1, or the *btaA* and *btaB* genes in the *T. pseudonana* genome, even though this diatom is known to produce betaine lipids in response to P deficiency [51]. In model systems, like *Arabidopsis*, SAM methyltransferase activity is an important step in betaine lipid biosynthesis, and there are 4 transcripts (PID: 3300, 12663, 30620 and 23867) with methyltransferase domains that are upregulated by P deficiency (Table S2). These warrant further study. Regardless, these findings underscore the molecular level regulation of the lipid replacement process, which is rapid [50], specific to P deficiency [51] and has not previously been examined in marine phytoplankton. Taken together, these data are consistent with a global restructuring of the cell-surface, the interface with which phytoplankton interact with their environment.

#### Modulation of glycolysis and translation

Several of the genes with coordinated regulation in the transcriptome and proteome are enzymes involved in glycolysis (Figure 2; Table S5). Work in higher plants and a green alga has demonstrated changes in glycolytic enzyme activities as a function of P deficiency, and it has been hypothesized that these changes are bypassing P dependent glycolytic reactions to recycle P and continue hexose-P conversion [56], [57]. The modulation of glycolysis related enzymes here (Figure 2), is permissive of a P deficiency induced glycolytic bypass mechanism in diatoms. This again illuminates an unrecognized aspect of the P stress response in this diatom, which may be common in other eukaryotic marine phytoplankton groups [58] and needs further study.

Coordination was also observed in the downregulated targets, with a downregulation of genes encoding ribosomal proteins observed in both transcript and protein datasets (Figure 2; Table S5), similar to that seen in the transcriptome of a marine cyanobacterium [59]. In other phytoplankton, RNA synthesis has been shown to be the single largest biochemical sink for P, accounting for about half of the total P uptake in the cyanobacteria *Prochlorococcus* [60]. Thus, under P deficiency there may be downregulation of ribosomal proteins and a repression of translation, to conserve P in rRNA. However this response may not be specific to P deprivation and may simply be a response to a reduced growth rate from any stressor. Further work that examines the degree of transcriptome and proteome coordination across multiple stressors and time points may help determine if P deficiency leads to a specific repression of translation.

Consistent with P deficiency leading to repression of translation, one of the more highly upregulated transcripts under P deficiency encodes a putative PUF family protein with a PUF RNA-binding domain (PID:31875) (Table S2). The PUF proteins characterized to date typically bind to 3′ UTR regions to repress gene expression by affecting mRNA translation or stability [61], [62], although their functions are diverse [63] and not characterized in any eukaryotic marine phytoplankton. Differential expression of this transcript was not detected in N, Fe and Si-limited *T. pseudonana* [19], suggesting that potential translational repression via this gene may not be a general nutrient stress response and may be specific to P deprivation.

Although many of the coordinated responses could be directly attributed to P acquisition and metabolism, glycolysis, or translation, there were a number of hypothetical genes responsive to P deficiency identified in both the transcriptome and the proteome (Table S5). As highlighted above there were also upregulated tags mapping to regions of the genome with no gene model. In the combined dataset of proteins and their corresponding transcripts (Figure 2, Table S5), the significantly upregulated gene set contained one predicted protein (PID:22734) with a kinase domain and a signal peptide indicating it is secreted. The rest of the hypothetical genes in this set have no discernable conserved domains or signal peptides. Two of these genes were detected by Mock et al. 2008 (PIDs: 3463, 22734), and shown to be downregulated by Si, Fe, and N limitation (PID:3463), or Si limitation (PID:22734), suggesting that their upregulation is unique to P metabolism. These non-modeled and hypothetical genes induced by P deficiency suggest that there are aspects of P physiology yet to be resolved in this diatom. These genes warrant further functional characterization for a truly comprehensive understanding of how diatoms scavenge P in the ocean.

### Conclusions

The diatom response to P deficiency involves a specific and multi-faceted remodeling of the transcriptome that is tightly coupled to the response of the proteome. There are also clear physiological changes in cellular P allocation patterns, enzyme activity, and lipid composition that are consistent with the transcriptome and proteome patterns. We identified differential regulation of a polyphosphate polymerase leading to a P deficiency-related increase in allocation of cellular P to polyphosphate. These data suggest that cycling and export of P as polyphosphate could be important in low or variable P systems where there is diatom P deficiency, in addition to high P environments where there is luxury uptake. This broader distribution of polyphosphate containing cells will influence the polyphosphate fueled formation and distribution of apatite containing sediments [8]. Although it is known that algae will induce phosphohydrolytic enzymes to acquire P from DOP in low P environments, to our knowledge this is the first work to demonstrate that diatoms have a cell-surface associated alkaline phosphatase, and may tightly couple hydrolysis and uptake. These results have implications for the competitive success of eukaryotic phytoplankton in low P systems and biogeochemical modeling efforts. Past work on DOP utilization has primarily focused on phosphomonoester hydrolysis and the enzyme alkaline phosphatase, but the strong induction of a diesterase suggests that recovery of phosphate from phosphodiester either from cell membranes, or exogenously, is a new critically important part of the diatom P stress response. Modeling the extent to which DOP supports primary production based on alkaline phosphatase activities alone, may thus be missing a large component of DOP hydrolysis. These observations, as well as the P regulation of a number of genes of unknown function, and the regulation of genes related to glycolysis and translation, provide new molecular-level insight into P stress responses in eukaryotic marine phytoplankton. In summary, these molecular data reveal that *T. pseudonana* has evolved a sophisticated response to P deficiency involving multiple biochemical strategies that are likely critical to its ability to rapidly respond to variations in environmental P availability, which should inform future field programs and biogeochemical modeling efforts.

## Methods

### Culture conditions and physiology measurements

*T. pseudonana* (Strain 1335 from the Provosoli-Guillard National Center for the Culture of Marine Phytoplankton (CCMP)) was grown in a modified f/2 medium [64] made from Sargasso Sea water. Macronutrients and vitamin B_12_, biotin, and thiamine solutions were treated with prepared Chelex-100 resin to remove trace metal contaminants followed by trace-metal clean syringe sterilization [65] to yield final nutrient concentrations of 882 µM NaNO_3_ and 106 µM Na_2_SiO_3_, and vitamin concentrations of 75 pM B_12_, 400 pM biotin, and 60 nM thiamine. The Fe concentration was also modified from f/2 to 400 nM. All conditions were run in triplicate at 14°C, in constant light (120 µmol photons m^−2^ s^−1^). Cells were grown with f/2 phosphorus concentrations (P-replete; 36 µM PO_4_) and with low phosphorus concentrations (P-deficient; 0.4 µM PO_4_). Growth was monitored daily with cell counts. Duplicate P-replete treatments were pooled and harvested in mid log phase, and triplicate P-deficient treatments were pooled, and also quickly harvested onto 2 µm filters at the onset of P depletion (Figure S1A). All RNA samples were snap frozen in liquid nitrogen. Replicate P-deficient cultures were refed to 36 µM phosphate at the onset of P depletion, and subsequently resumed growth (Figure S1A). Additional growth studies were performed as described above substituting glycerophosphate and adenosine monophosphate at 36 µM, for the phosphate in the medium (Figure S1B).

Alkaline phosphatase activity was assayed on cells using a fluorometric method described elsewhere [12]. Labeling of alkaline phosphatase activity on whole cells was performed using enzyme labeled fluorescence (ELF) with previously reported methods [13]. Confocal microscopy of ELF-labeled cells was performed at the Marine Biological Laboratories using a Zeiss Inverted LSM 710, and a spectral detector to isolate the chlorophyll (680 nm) and ELF-97 product (535 nm) signals after Dyhrman and Palenik (1999). To assay diesterase activity cells were harvested via gentle filtration and resuspended into 2 mL of Buffer A [43] with 5.0 mM bis-4(nitrophenyl)phosphate. Absorbance was measured at 410 nm relative to substrate only and cell only controls on a spectrophotometer. Polyphosphate was detected using solid state ^31^P nuclear magnetic resonance spectroscopy on cells collected onto 0.2 µm polycarbonate filters and analyzed as described elsewhere [66], [67].

### Transcriptome analysis

Transcriptome profiling was completed using a tag-seq profiling approach by Illumina Inc. Briefly, equal volumes from duplicate P-replete cultures, and triplicate P-deficient cultures were pooled prior to filtration. RNA was extracted from 5.40×10^8^ cells and 3.51×10^8^ cells, from the P-replete and P-deficient samples, respectively, using Qiagen's RNeasy Midi kit according to the manufacturer's instructions with the following exceptions; cells were lysed using 0.5 mm zirconia/silica beads (BioSpec) along with the lysis buffer solution and vortexed until the solution looked homogenous (2–3 min). The lysis solution was then put over Qiashredder columns (Qiagen) to aid in the removal of any large cell wall material that could clog the spin columns. To aid in the removal of DNA, an on-column DNase digestion was performed during the extraction using the RNase-free DNase Set (Qiagen) according to the manufacturer's instructions. Roughly 11 µg of P-replete and 8 µg of P-deficient total RNA was sent to Illumina and converted to cDNA. Illumina library construction included a polyA selection and digestion with NlaIII. This resulted in 21 bp sequence reads called tags that should map to the most 3′ NlaIII site in each transcript, but often map to alternative sites likely due to incomplete digestion during library construction.

Herein, tag-seq detected a total of 781,210 unique tags across both libraries generated from P-replete and P-deficient cultures (Table S1). The data are deposited in NCBI's Gene Expression Omnibus and are accessible through GEO Series accession number GSE28134. The 34.5 Mb *T. pseudonana* genome sequence [30] was used for tag annotation. Tag to gene mapping was performed through a pipeline designed by Genesifter Inc. In brief, the tag including the NlaIII site was mapped to the genome sequence data available at NCBI (AAFD00000000), requiring a 100% identity. Tags that mapped to more than one location were removed from the analysis. Of the roughly 12 million total sequence reads for each treatment, approximately 75% could be mapped to the genome (Table S1), a high number in part because of the presence of many unique tags with high counts. This percentage is similar to that observed for a DGE study in *Chlamydomonas reinhardtii* [29]. Approximately 10% of the unique tags could be mapped. This percentage of tag to genome mapping is somewhat lower than other studies with lower coverage [32]. For organisms with the vast majority of genes in their genome unmapped and uncharacterized, such as *T. pseudonana*, it is common to have relatively low percentages of tags mapping to the genome. Unique tags may not map because of single nucleotide polymorphisms (biological or introduced by sequencing error), because of incorrectly assigned intron/exon boundaries, and because tags map to 3′ UTRs that were not included in the predicted gene models among other possibilities.

Tag-seq, like Long-SAGE, detects both sense and antisense transcripts. Here, tags frequently mapped to both the forward and reverse strands of a given NlaIII site. For the differential expression analysis, all sense tags mapping within a given gene model were pooled, and antisense tags were excluded. Tag frequency was normalized to the total tags sequenced in a given library and the frequencies are reported in tags per million (tpm). Dynamic range was robust with gene model counts ranging from 1 to >5600 tags per million (tpm). Statistical significance in differential expression was determined by computing the posterior mean of the log ratio of proportions over the two conditions using an empirical Bayes method (ASC) [25]. In brief, the expectation of log fold change given the observed counts for each tag is estimated, and a standardized score is computed by dividing the estimated log ratio by a shrunken standard error estimate representing the biological variation. Transcripts, or tags, with a local false discovery rate (FDR)<0.05 are reported as differentially expressed (Table S2). The ASC statistic is designed for high density, high coverage datasets of transcriptional patterns generated with high throughput sequencing and is less biased by high tag counts than the Z score and Fisher test [25].

### Proteome analysis

Biomass from frozen filter samples was scraped into tubes with a clean spatula then resuspended in 600 µL B-PER reagent (Thermo Scientific, Rockford, IL) supplemented with 0.5 mM EDTA and protease inhibitor 1mMphenylmethanesulfonylfluoride. Samples were vortexed for 1 minute and incubated at room temperature for 20 minutes then chilled on ice for 10 minutes. The cells were then sonicated with a microtip on ice for 1 minute at constant duty cycle, with the temperature not exceeding 28°C. Samples were centrifuged for 30 minutes at 14,100 RCF and 4°C, and supernatants were transferred and precipitated overnight in 50% acetone 50% methanol with 0.5 mM HCl at −20°C. Precipitated protein was collected by centrifugation at 14,100 RCF for 30 minutes at 4°C and dried by speed vacuum at room temperature. Protein was resuspended in B-Per/EDTA/PMSF extraction buffer, gently mixed and incubated for 30 minutes at room temperature and splits were combined. Aliquots were taken for protein determination by DC assay using bovine serum albumin as a protein standard (BioRad Inc., Hercules CA). Proteins were stored at −80°C until digestion.

Protein was then digested following the tube gel digestion procedure [68] with some modifications. Briefly, samples were immobilized in 15% acrylamide in pH 7.5 Tris buffer, incubated twice with 10% acetic acid and 50% ethanol for 20 min and 1 h, then with 10% acetic acid and 50% methanol for 2 h at room temperature and mixing at 350 RPM, decanting between. Gel samples were cut into ∼1 mm^3^ pieces and incubated twice with 50% acetonitrile 50% 25 mM ammonium bicarbonate solution for 1 h and again overnight, shaking at 350 RPM at 16°C and decanting between. Proteins immobilized within the gel were successively reduced with 10 mM dithiothreitol (DTT) at 56°C for 1 h, decanted, and alkyated with 30 mM iodoacetamide for 1 h at room temperature, washed in 25 mM ammonium bicarbonate for 20 minutes and twice with 100% acetonitrile for 10 minutes, and dried for 20 minutes by speed vacuum. Gel pieces were rehydrated and digested with a solution of 10 µg trypsin in 25 mM ammonium bicarbonate for 16 h at 37°C (1∶20 ratio trypsin to total protein, Promega Gold Mass Spectrometry Grade, Promega Inc., Madison WI). The peptides were extracted by successive additions of 50 percent acetonitrile (Fisher Optima) with 5% formic acid (Michrom Ultra Pure). The extracted peptides were combined and concentrated by speed vacuum for about three hours to less than 20 µL, diluted with 2 percent acetonitrile and 0.1 percent formic acid in water (Fisher Optima) and stored at −80°C.

The protein digestions were analyzed using a peptide Cap Trap in-line with a reversed phase Magic C18 AQ column (0.2×150 mm, 3 µm particle size, 200 Å pore size, Michrom Bioresources Inc. Auburn CA) on a Paradigm MS4 HPLC system (Michrom Bioresources Inc.) at a flow rate of 2 µl minute^−1^. A LTQ linear ion trap mass spectrometer (Thermo Scientific Inc. San Jose CA) was used with an ADVANCE electrospray source (Michrom Bioresources Inc.). The chromatography consisted of a hyperbolic gradient from 5% buffer A to 95% buffer B for 300 minutes, where A was 0.1% formic acid (Michrom Ultra Pure) in water (Fisher Optima) and B was 0.1% formic acid in acetonitrile (Fisher Optima). The mass spectrometer was set to perform MS/MS on the top 7 ions using data-dependent settings and a dynamic exclusion window of 30 s. Ions were monitored over the range of 400–2000 m/z.

The mass spectra collected in this study were searched using SEQUEST (Bioworks version 3.3, Thermo Inc., San Jose CA) using an *in silico* tryptic peptide database assembled from the JGI modeled gene set (Thaps 3.0) (Thaps3_bd_unmapped_GeneModels_FilteredModels1_aa.fasta.gz and Thaps3_chromosomes_geneModels_FilteredModels2_aa.fasta.gz) with the addition of NCBI *T. pseudonana* chloroplast (EF067921) and mitochondria (DQ186202) genomes and a reversed ‘decoy’ version of these databases for false discovery rate analysis. SEQUEST parameters were set at 30% ions required per peptide, DCN of 0.1, Xcorr vs CS 1.9, 2.4, 2.9, and 1e-3 protein probability, with a false positive rate of 0.96% [35]. Changes in each protein's relative abundances across treatments were calculated using normalized spectral counts within Scaffold (Proteome Software V3.0; protein identification probabilities of 99.0% and requiring at least two tryptic peptide identifications per protein for 1264 protein identifications) (Table S4). Database search results were further processed using the PeptideProphet statistical model [69] within Scaffold 3.0 (Proteome Software Inc., Portland OR). Relative protein abundance was determined using Scaffold 3.0 for normalized spectral counting software operating on a 64bit Ubuntu Linux workstation. Spectral counts are normalized across samples in each experiment in Scaffold, including technical replicates, to allow comparison of relative protein abundance. Comparison of technical triplicate injections on the LC-MS demonstrates robust relative quantitation of the abundant proteins in the *T. pseudonana* proteome, with repeat injections showing similar spectral count values for each protein (Figure S2). Proteins discussed as ‘differentially expressed’ were determined by the Fisher exact test with *p*<0.05 [70] (Table S4).

## Supporting Information

Figure S1 **Growth experiments.** (A) Growth of experimental *T. pseudonana* CCMP 1335 cultures in different treatments. Cells were inoculated into triplicate treatments containing 36 µM phosphate (+P; P-replete), or 0.4 µM phosphate (-P; P-deficient) and harvested at 100 hrs for experimental RNA as indicated (arrow). At the 100 hr time point the 0.4 µM phosphate treatments were split and half were re-fed with phosphate to 36 µM (P re-addition), to confirm that the –P treatments were P-deficient. (B) Triplicate *T. pseudonana* CCMP 1335 cultures in different phosphorus treatments. Cells were inoculated into triplicate treatments containing no added phosphorus (no P), or 36 µM phosphate (DIP), adenosine monophosphate (AMP), or glycerophosphate (GLY-P). Growth was assessed by relative fluorescence daily.
(PDF)

Figure S2 **Technical replication of the proteome spectral counts.** A comparison of protein extraction replicates from two pooled samples from P-deficient cultures, showing excellent reproducibility where values adhere to a 1∶1 line. Both samples were pooled from equal amounts of two biological replicates, each filtered onto a 2 micron filter and extracted in parallel. The inset is an expansion of the data at the origin to visualize the lower abundance proteins.
(PDF)

Table S1 **Transcript and protein statistics for P-replete and P-deficient** ***T. pseudonana*** **.**
(DOC)

Table S2 **Detected transcripts, their annotation, abundance, and statistical significance.**
(XLS)

Table S3 **The top 50 most highly upregulated sequence reads (tags) and their annotation information.**
(XLS)

Table S4 **Peptides detected in P-replete and P-deficient** ***T. pseudonana*** **.**
(XLS)

Table S5 **Combined transcript and protein abundance data for P-replete and P-deficient** ***T. pseudonana***.
(XLS)
