# Supplementary figures and images for: The Transcriptome and Proteome of the Diatom Thalassiosira pseudonana Reveal a Diverse Phosphorus Stress Response
Source: PLoS One. 2012 Mar 29;7(3):e33768. doi: 10.1371/journal.pone.0033768 (PMC3315573; doi:10.1371/journal.pone.0033768)

A

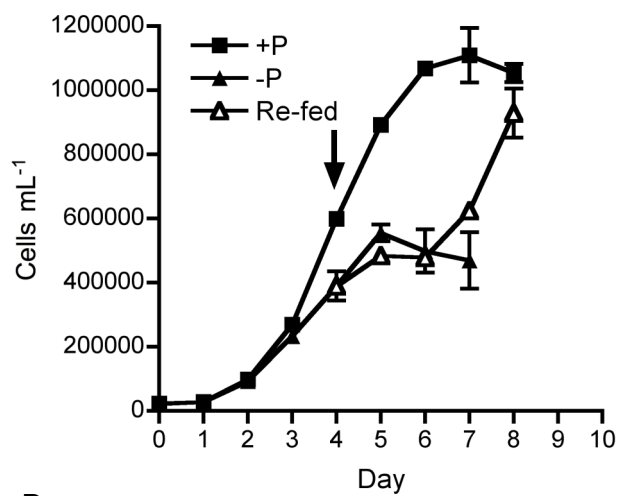

B

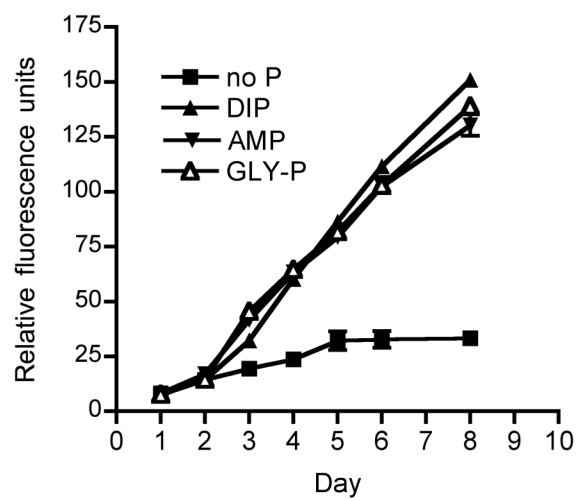

Supplement: Figure S1 — Growth experiments. (A) Growth of experimental T. pseudonana CCMP 1335 cultures in different treatments. Cells were inoculated into triplicate treatments containing 36 µM phosphate (+P; P-replete), or 0.4 µM phosphate (-P; P-deficient) and harvested at 100 hrs for experimental RNA as indicated (arrow). At the 100 hr time point the 0.4 µM phosphate treatments were split and half were re-fed with phosphate to 36 µM (P re-addition), to confirm that the –P treatments were P-deficient. (B) Triplicate T. pseudonana CCMP 1335 cultures in different phosphorus treatments. Cells were inoculated into triplicate treatments containing no added phosphorus (no P), or 36 µM phosphate (DIP), adenosine monophosphate (AMP), or glycerophosphate (GLY-P). Growth was assessed by relative fluorescence daily. (PDF) [file pone.0033768.s001.pdf]

Figure S2.

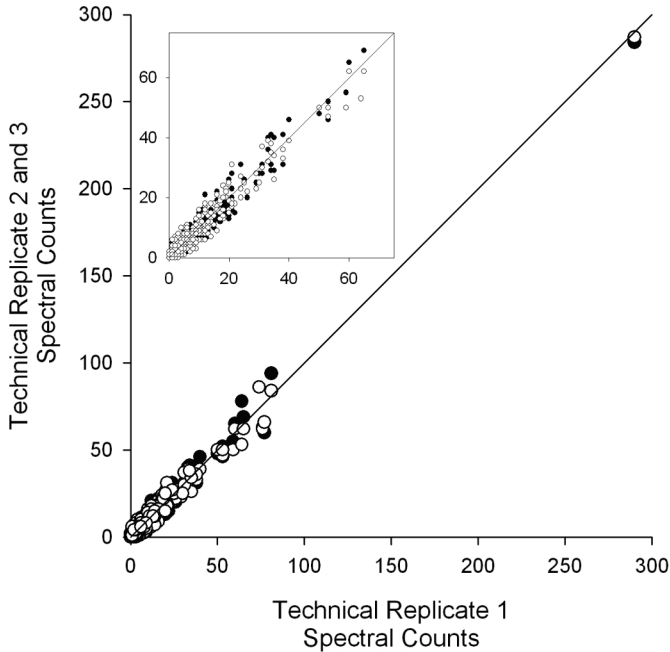

Supplement: Figure S2 — Technical replication of the proteome spectral counts. A comparison of protein extraction replicates from two pooled samples from P-deficient cultures, showing excellent reproducibility where values adhere to a 1∶1 line. Both samples were pooled from equal amounts of two biological replicates, each filtered onto a 2 micron filter and extracted in parallel. The inset is an expansion of the data at the origin to visualize the lower abundance proteins. (PDF) [file pone.0033768.s002.pdf]
