# Supplementary material for: The Transcriptome and Proteome of the Diatom Thalassiosira pseudonana Reveal a Diverse Phosphorus Stress Response
Source: PLoS One. 2012 Mar 29;7(3):e33768. doi: 10.1371/journal.pone.0033768 (PMC3315573; doi:10.1371/journal.pone.0033768)
Supplement: Table S1 — Transcript and protein statistics for P-replete and P-deficient T. pseudonana . (DOC) [file pone.0033768.s003.doc]

**Table S1. Library statistics for the *T. pseudonana*** transcriptome and proteome data.

| **Library** | **No. of reads** | **% mapped reads^1^** | **No. gene models^2^** | **No. of regulated unmapped reads ^3^** | **No. of regulated gene models ^3^** | **No. of detected proteins** | **No. of more abundant proteins^4^** |
| --- | --- | --- | --- | --- | --- | --- | --- |
| P-replete | 12,335,963 | 77% | 9,380 | - | - | 1036 | 57 |
| P-deficient | 13,431,745 | 76% | 9,519 | 1382 | 318 | 941 | 79 |

^1^ No basepair mismatches were allowed for tag mapping, for each library roughly 50% of tags mapped to gene models, the remainder to intergenic space.

^2^ Computationally predicted gene models with at least one tag mapped at 100%. The total coverage on unique gene models across both libraries is 9,572.

^3^ The total number significantly differentially expressed transcripts (up and down) were detected by Analysis of Sequence Counts with a local false discovery rate (FDR) <0.05

^4^ The total number significantly differentially abundant proteins (up and down) were detected by a Fisher exact test with *p*<0.05.
